# Exploring the link between cancer policies and cancer survival: a comparison of seven countries

**DOI:** 10.1093/eurpub/ckac129.166

**Published:** 2022-10-25

**Authors:** E Nolte, M Morris, S Landon, M McKee, M Seguin, J Butler, M Lawler

**Affiliations:** 1 Department of Health Services Research and Policy, LSHTM, London, UK; 2 The Royal Marsden Hospital, London, UK; 3 Cancer Research UK, London, UK; 4 Patrick G Johnston Centre for Cancer Research, Queen's University Belfast, Belfast, UK

## Abstract

**Background:**

Disparity in cancer survival across countries has been linked to variation in cancer policy delivery but there is lack of empirical evidence for this association. We traced the evolution of cancer policies in 20 jurisdictions in Australia, Canada, Denmark, Ireland, Norway, New Zealand and the UK since 1995 and present the findings of an exploratory analysis linking cancer policy consistency to cancer survival.

**Methods:**

We systematically searched and analysed national/regional cancer plans and strategies, mapping timelines of cancer policy evolution. For 10 jurisdictions, evidence was synthesised into five categories: oversight function; cancer plan; implementation plan; budget for plan implementation; and evaluation. We assigned scores evaluating whether a category was present or absent, and weighted scores for consistency. Summed scores were correlated with trends in survival from seven cancers between 1995-2014.

**Results:**

All ten jurisdictions had implemented a high-level structure overseeing, steering or delivering cancer control policies (1995 - 2014); all had also published at least one major cancer plan. There was great variation in oversight mechanisms, ranging from institutionalising cancer control (New South Wales, Ontario) to cancer steering groups or taskforces (Denmark, Northern Ireland, Wales). Frequency and consistency of cancer plans also varied, from a succession of plans that build on each other (Denmark, New South Wales, Ontario) to the publication of isolated plans (New Zealand, Northern Ireland). We found a positive, albeit weak, correlation of cancer policy consistency and improvements in survival over time for six of the seven cancers.

**Conclusions:**

Jurisdictions that have implemented consistent cancer control policies over time tended to be more successful in improving survival for a wide range of cancers. Our findings can help guide policymakers seeking approaches and frameworks to improve cancer services and, ultimately, cancer outcomes.

**Key messages:**

• Sustained and consistent strategic cancer planning and investment are crucial for ensuring better patient outcomes, and this requires strong and sustained commitment at all levels.

• The findings can help guide policymakers seeking approaches and frameworks to improve cancer services and, ultimately, cancer outcomes.

